# A Survey of Pyridoxal 5′-Phosphate-Dependent Proteins in the Gram-Positive Model Bacterium *Bacillus subtilis*

**DOI:** 10.3389/fmolb.2019.00032

**Published:** 2019-05-10

**Authors:** Björn Richts, Jonathan Rosenberg, Fabian M. Commichau

**Affiliations:** Department of General Microbiology, University of Goettingen, Göttingen, Germany

**Keywords:** vitamin B6, PLP-ome, amino transferase, metabolic engineering, toxicity

## Abstract

The B6 vitamer pyridoxal 5′-phosphate (PLP) is a co-factor for proteins and enzymes that are involved in diverse cellular processes. Therefore, PLP is essential for organisms from all kingdoms of life. Here we provide an overview about the PLP-dependent proteins from the Gram-positive soil bacterium *Bacillus subtilis*. Since *B. subtilis* serves as a model system in basic research and as a production host in industry, knowledge about the PLP-dependent proteins could facilitate engineering the bacteria for biotechnological applications. The survey revealed that the majority of the PLP-dependent proteins are involved in metabolic pathways like amino acid biosynthesis and degradation, biosynthesis of antibacterial compounds, utilization of nucleotides as well as in iron and carbon metabolism. Many PLP-dependent proteins participate in *de novo* synthesis of the co-factors biotin, folate, heme, and NAD^+^ as well as in cell wall metabolism, tRNA modification, regulation of gene expression, sporulation, and biofilm formation. A surprisingly large group of PLP-dependent proteins (29%) belong to the group of poorly characterized proteins. This review underpins the need to characterize the PLP-dependent proteins of unknown function to fully understand the “PLP-ome” of *B. subtilis*.

## Introduction

**Figure 1:**
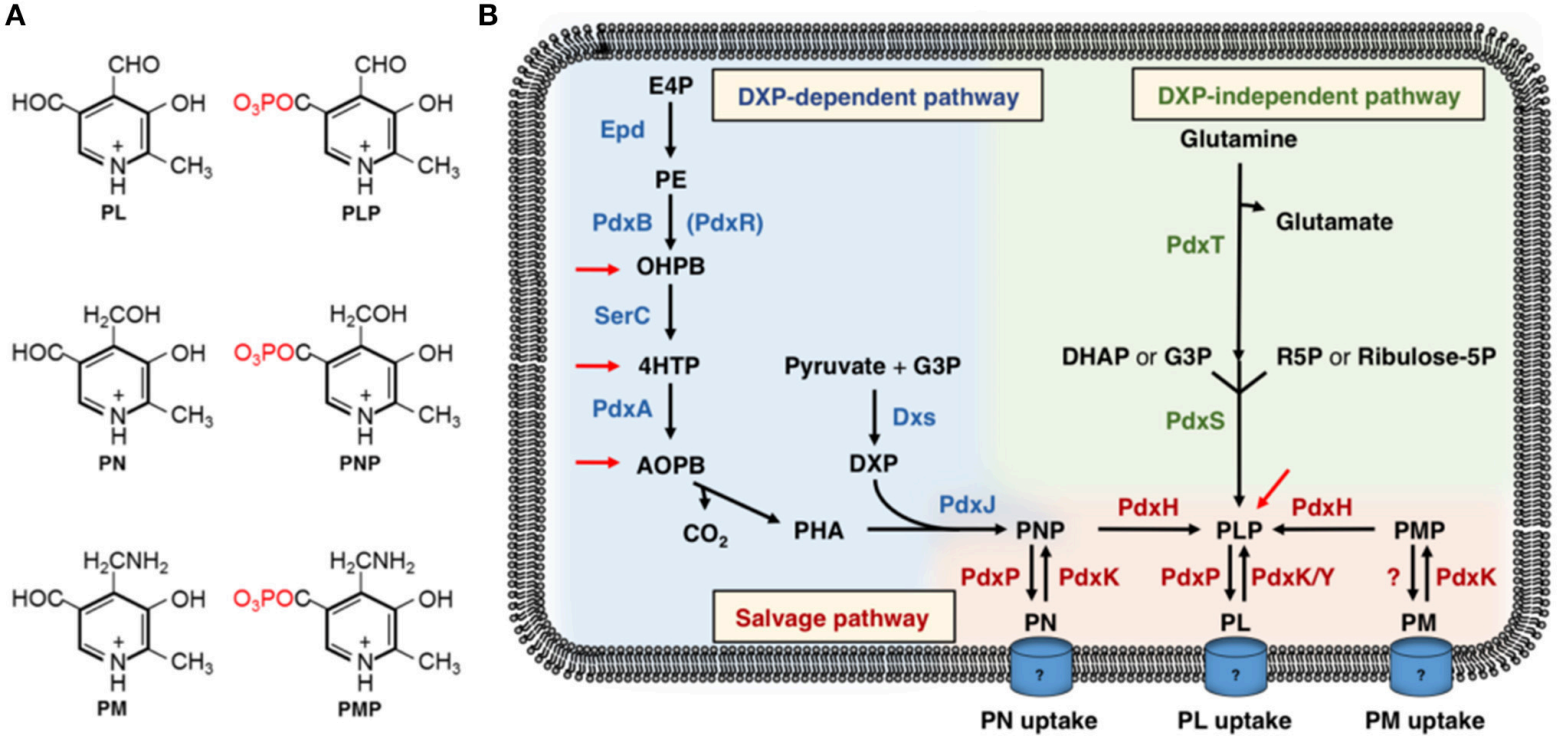
**(A)** The B6 vitamers: pyridoxal (PL), pyridoxal 5′-phosphate (PLP), pyridoxine (PN), pyridoxine 5′-phosphate (PNP), pyridoxamine (PM), and pyridoxamine 5′-phosphate (PMP). **(B)** The deoxyxylulose 5-phosphate (DXP)-dependent and DXP-independent vitamin B6 biosynthetic routes and the salvage pathway for the interconversion of the B6 vitamers. Epd, erythrose 4-phosphate dehydrogenase; PdxB (PdxR), 4-phosphoerythronate dehydrogenase; SerC, 3-phosphoserine aminotransferase; PdxA, 4-phosphohydroxy-L-threonine dehydrogenase; PdxJ, PNP synthase; Dxs, 1-deoxyxylulose 5-phosphate synthase; PdxH, PNP oxidase; PdxS (PLP synthase subunit), and PdxT (glutaminase subunit) form the PLP synthase complex; PdxK, PL kinase present in *B. subtilis* and *E. coli*; PdxY, PL kinase present in *E. coli*. PdxK from *B. subtilis* has PN, PL, and PM kinase activity (see text). E4P, erythrose 4-phosphate; 4PE, 4-phosphoerythronate; OHPB, 2-oxo-3-hydroxy-4-phosphobutanoate; 4HTP, 4-hydroxy-threonine; AOPB, 2-amino-3-oxo-4-(phosphohydroxyl)-butyrate; PHA, 3-phosphohydroxy-1-aminoacetone; DXP, deoxyxylulose-5-phosphate; G3P, glyceraldehyde-3-phosphate; DHAP, dihydroxyacetonephosphate; R5P, Ribose-5-phosphate. Red arrows indicate the steps where promiscuous enzymes may feed into the DXP-dependent and DXP-independent vitamin B6 biosynthetic pathways (Kim J. et al., 2010; Oberhardt et al., 2016; Thiaville et al., 2016; Rosenberg et al., 2018).

The term “vitamin B6” collectively designates the vitamers pyridoxal (PL), pyridoxine (PN), and pyridoxamine (PM), and the respective phosphate esters pyridoxal 5′-phosphate (PLP), pyridoxine 5′-phosphate (PNP), and pyridoxamine 5′-phosphate (PMP) (György, 1956; Rosenberg, 2012) (Figure 1A). Since vitamin B6 is an essential micronutrient component in the diet of mammals, it is of commercial interest for the pharmaceutical and the food industry (Domke et al., 2005; Fitzpatrick et al., 2007, 2010; Eggersdorfer et al., 2012; Kraemer et al., 2012; Rosenberg et al., 2017; Acevedo-Rocha et al., 2019). As yet, the B6 vitamers are chemically synthesized via different routes (Pauling and Weimann, 1996; Kleemann et al., 2008; Eggersdorfer et al., 2012). Since chemical synthesis requires the usage of expensive and/or toxic chemicals, the shift from chemical synthesis to sustainable fermentation technologies using microorganisms is of great interest (Rosenberg et al., 2017; Acevedo-Rocha et al., 2019). So far, the microbial vitamin B6 production processes could not replace chemical production processes (Commichau et al., 2014, 2015; Rosenberg et al., 2017, 2018; Acevedo-Rocha et al., 2019).

PLP is a co-factor for many proteins and enzymes (Jansonius, 1998; Christen and Mehta, 2001; Eliot and Kirsch, 2004; Phillips, 2015). About 1.5% of the genes of free-living prokaryotes encode PLP-dependent proteins and over 160 enzymes with different catalytic activities require vitamin B6 as a co-factor (about 4% of all described catalytic activities) (Percudani and Peracchi, 2003, 2009). Certainly, novel PLP-dependent proteins and enzymes will be identified and characterized in the future because the number of sequenced genomes is increasing (https://www.ncbi.nlm.nih.gov/genome/browse/#!/overview/). The majority of the PLP-dependent enzymes are involved in amino acid metabolism (John, 1995; Eliot and Kirsch, 2004). Some enzymes catalyzing decarboxylation and racemization reactions, cleavage of *C*_*a*_*-C*_*b*_ bonds, α-elimination and replacement as well as β- and γ-elimination or replacement reactions also require PLP as a co-factor. Moreover, PMP and PM serves as co-factors for enzymes of deoxysugar and amino acid biosynthetic pathways, respectively (Burns et al., 1996; Mehta and Christen, 2000; Yoshikane et al., 2006; Romo and Liu, 2011). PLP also modulates the activity of DNA-binding transcription factors in eukaryotes and prokaryotes (Oka et al., 2001; Belitsky, 2004a, 2014; Huq et al., 2007; El Qaidi et al., 2013; Tramonti et al., 2015, 2017; Suvorova and Rodionov, 2016). Moreover, vitamin B6 is implicated in oxidative stress responses (Bilski et al., 2000; Mooney et al., 2009; Mooney and Hellmann, 2010; Vanderschuren et al., 2013; Moccand et al., 2014). Thus, vitamin B6 fulfills a variety of vital functions in different cellular processes (Parra et al., 2018).

## *De novo* Synthesis Of Vitamin B6

Two pathways for *de novo* PLP synthesis are currently known (Figure 1B) (Mittenhuber, 2001; Tanaka et al., 2005; Fitzpatrick et al., 2007, 2010; Rosenberg et al., 2017). The deoxyxylulose-5-phosphate (DXP)-dependent vitamin B6 biosynthesis pathway was identified in the Gram-negative model bacterium *Escherichia coli* and consists of two branches and seven enzymatic steps. The first three enzymes Epd, PdxB, and SerC of the longer branch convert a pentose phosphate pathway intermediate to 4-phosphohydroxy-L-threonine (4HTP) (Figure 1B) (Zhao et al., 1995; Drewke et al., 1996; Boschi-Muller et al., 1997; Tazoe et al., 2006; Rudolph et al., 2010). Next, PdxA converts 4HTP to 2-amino-3-oxo-4-(phosphohydroxy)butyric acid, which undergoes spontaneous decarboxylation to 3-phosphohydroxy-1-aminoacetone (Cane et al., 1998; Laber et al., 1999; Sivaraman et al., 2003). The PNP synthase PdxJ produces the B6 vitamer PNP from 3-phosphohydroxy-1-aminoacetone and DXP, of which the latter substrate is generated by the DXP synthase Dxs from glyceraldehyde 3-phosphate and pyruvate in the short branch of the DXP-dependent vitamin B6 pathway (Figure 1B) (Takiff et al., 1992; Sprenger et al., 1997; Cane et al., 1999; Laber et al., 1999). The PNP oxidase PdxH catalyzes the final step yielding in the biologically most-relevant B6 vitamer PLP (Zhao and Winkler, 1995). The DXP-dependent vitamin B6 pathway is present in α- and γ-proteobacteria (Mittenhuber, 2001; Tanaka et al., 2005). Recently, it has been shown that bacteria possess promiscuous enzymes that may feed into the DXP-dependent pathway and bypass a block in pyridoxal-5′-phosphate synthesis (Figure 1B) (Kim J. et al., 2010; Kim and Copley, 2012; Smirnov et al., 2012; Oberhardt et al., 2016; Thiaville et al., 2016; Zhang et al., 2016; Rosenberg et al., 2018). The hybrid pathways consisting of enzymes of native and non-native vitamin B6 pathways and of promiscuous enzymes may be improved by metabolic engineering to enhance production of B6 vitamers (Rosenberg and Commichau, 2019).

The DXP-independent vitamin B6 biosynthetic pathway involves only the PdxST enzyme complex (Ehrenshaft and Daub, 2001; Belitsky, 2004b; Burns et al., 2005; Raschle et al., 2005; Strohmeier et al., 2006). PdxT is a glutaminase that hydrolyses glutamine into glutamate and ammonium, of which the latter serves as a substrate to the PLP synthase PdxS (Belitsky, 2004b). The PdxS subunit generates PLP from ammonium together with either ribulose 5-phosphate or ribose 5-phosphate and with either G3P or dihydroxyacetone phosphate (Figure 1B). Many organisms possess a salvage pathway for the interconversion of the B6 vitamers (Figure 1B) (Fitzpatrick et al., 2007; di Salvo et al., 2011). For instance, specific B6-vitamer kinases can phosphorylate PN, PM and PL into their respective phosphate esters (White and Dempsey, 1970; Yang et al., 1996, 1998; di Salvo et al., 2004; Park et al., 2004). Those organisms carrying only salvage pathways have to take up B6 vitamers. So far, only few vitamin B6 transporters have been in described in eukaryotes (Stolz and Vielreicher, 2003; Szydlowski et al., 2013). The accumulation of PLP to toxic levels can be prevented by dephosphorylation and export of PL. Indeed, bacteria like *E. coli* and *Sinorhizobium meliloti* synthesize a phosphatase for dephosphorylation of PNP and PLP (Tazoe et al., 2005; Nagahashi et al., 2008; Sugimoto et al., 2017).

## Vitamin B6 Metabolism in *Bacillus subtilis*

The Gram-positive model bacterium *Bacillus subtilis* relies on the PdxST enzyme complex to synthesize the B6 vitamer PLP (Pflug and Lingens, 1978; Sakai et al., 2002b; Belitsky, 2004b; Burns et al., 2005). The *B. subtilis* PdxST enzyme complex has been biochemically and structurally studied (Raschle et al., 2005; Zhu et al., 2005; Strohmeier et al., 2006; Wallner et al., 2009; Smith et al., 2015). In many Gram-positive bacteria, the expression of the *pdxST* genes and the genes encoding kinases that phosphorylate the B6 vitamers PM, PN, and PL, are regulated by MocR-like DNA-binding transcription factors (Jochmann et al., 2011; El Qaidi et al., 2013; Belitsky, 2014; Liao et al., 2015; Tramonti et al., 2015; Suvorova and Rodionov, 2016). In *B. subtilis* the *pdxST* genes are not subject to transcriptional regulation (Nicolas et al., 2012).

*B. subtilis* must possess an uptake system and a kinase for the B6 vitamer PL because exogenously supplied PL relieves PLP auxotrophy of a *pdxST* mutant (Belitsky, 2004b; Commichau et al., 2014). The uptake system and the kinase are not specific for PL because the overexpression of the *E. coli pdxH* PN(P) oxidase gene in a *B. subtilis pdxST* mutant enabled the bacteria to synthesize PLP from exogenous PN (Commichau et al., 2014). While the PL and PN uptake system remains to be identified in *B. subtilis*, the kinase phosphorylating the B6 vitamers PL, PM, and PN is known (Park et al., 2004; Newman et al., 2006a,b). It is interesting to note that the *B. subtilis* PL kinase PdxK is phylogenetically related to HMPP ribokinases converting 4-amino-5-hydroxymethyl-2-methylpyrimidine (HMP) to 4-amino-5-hydroxymethyl-2-methylpyrimidine phosphate (HMPP) and HMPP to HMPP phosphate, a precursor of thiamine biosynthesis (Mizote et al., 1999; Newman et al., 2006b). In the future, it will be interesting to elucidate whether exogenously supplied PL controls the activity of the PdxST enzyme complex in *B. subtilis* to prevent the accumulation of PLP to toxic levels (see below).

In contrast to the enzymes of the DXP-dependent vitamin B6 pathway from *E. coli*, the PdxST enzyme complex from *B. subtilis* is rather slow (Rosenberg et al., 2017). Therefore, a heterologous DXP-dependent vitamin B6 pathway has been introduced into *B. subtilis* for producing the B6 vitamer PN (Commichau et al., 2014, 2015). The fact that the engineered *B. subtilis* strains synthesized significant amounts of PN, which was detectable in the culture supernatant, suggests that the bacteria might possess a PNP phosphatase and an export system for PN. Recently, the PLP phosphatase YbhA has been identified in *E. coli* (Sugimoto et al., 2017). YbhA shows about 31% overall sequence identity with the YitU protein from *B. subtilis*. Even though it has been shown that YitU is a HAD phosphatase having a minor activity in dephosphorylating the riboflavin precursor 5-amino-6-ribitylamino-2,4(1H,3H)-pyrimidinedione 5′-phosphate (Sarge et al., 2015), it will be interesting to test whether the protein may act as a PNP/PLP phosphatase.

## Toxicity of Vitamin B6 and Pathway Intermediates

PLP can be toxic for the cell because the reactive 4′-aldehyde moiety of the B6 vitamer forms covalent adducts with other compounds and PLP-independent proteins containing thiol or amino groups. For instance, PLP was shown to inhibit enzymes that are involved in DNA metabolism and in central carbon metabolism in eukaryotes (Mizushina et al., 2003; Vermeersch et al., 2004; Lee et al., 2005). Moreover, the modification of the *E. coli* initiation factor 3, the adenylsuccinate synthetase and the PL kinase by PLP results in activity loss (Ohsawa and Gualerzi, 1981; Dong and Fromm, 1990; Ghatge et al., 2012). Recently, it has been shown that the addition of vitamin B6 to the *E. coli* wild type strain BW25113 and an *E. coli* mutant strain lacking the ZipA cell division protein affects multiple metabolic pathways, which are involved in amino acid biosynthesis (Vega and Margolin, 2017). Thus, excess of PLP affects different cellular processes. PLP is also prone to damage either due to side reactions that are catalyzed by promiscuous enzymes or due to spontaneous chemical reactions (Linster et al., 2013). In fact, the B6 vitamers PLP and PMP were identified as members of the 30 most damage-prone metabolites (Lerma-Ortiz et al., 2016). However, given the fact that PLP is required for optimal growth in little amounts, the essential co-factor can be synthesized at a minimal necessary rate (Hartl et al., 2017). The low requirement of PLP and its low cellular concentration prevent perturbation of other essential processes in the cell.

In the past years, several attempts have been made to engineer bacteria for the overproduction of the B6 vitamers PL and PN (Rosenberg et al., 2017). In contrast to PL(P), PN(P) is less toxic for living cells (see above; Commichau et al., 2014). Therefore, the DXP-dependent seems to be a promising pathway for engineering bacteria for vitamin B6 overproduction. However, it has also been shown that intermediates of the DXP-dependent pathway can be highly toxic for bacteria. For instance, the erythrose-4-phosphate dehydrogenase Epd generates the DXP-dependent vitamin B6 pathway intermediate 4-phosphoerythronate (4PE), which is required in low amounts for PLP biosynthesis, is toxic for the cells when overproduced (Sachla and Helmann, 2019). Eukaryotic cells do have a phosphatase that hydrolyzes and detoxifies 4PE that is also mistakenly generated by the glycolytic enzyme glyceraldehyde-3-phosphate dehydrogenase (Beaudoin and Hanson, 2016; Collard et al., 2016). 4PE inihibits the 6-phosphogluconate dehydrogenase from the pentosephosphate pathway (Collard et al., 2016). Recently, it has been demonstrated that 4PE also inhibits the *B. subtilis* 6-phosphogluconate dehydrogenase GndA (Sachla and Helmann, 2019). In this organism, 4PE is detoxified by the GTPase CpgA, which is a checkpoint protein known to be involved in ribosome assembly (Campbell et al., 2005). It will be interesting to assess whether 4PE also inhibits the 6-phosphogluconate dehydrogenase in *E. coli* because the bacterium does not possess a CpgA homolog. However, the accumulation of 4PE to toxic levels does not seem to be problematic in *E. coli* because 4PE can be produced in only small amounts that are sufficient for *de novo* synthesis of PLP. The intermediate 4HTP from the DXP-dependent vitamin B6 pathway is also inhibits bacterial growth. 4HTP interferes with biosynthesis of threonine and isoleucine in *E. coli* and *B. subtilis* (Drewke et al., 1993; Farrington et al., 1993; Commichau et al., 2014, 2015; Rosenberg et al., 2016). The toxicity of the pathway intermediates may explain why it is difficult to engineer bacteria that stably express the genes of the DXP-dependent vitamin B6 pathway (Commichau et al., 2014). The understanding of the metabolite toxicity is crucial for the rational design and engineering of bacteria overproducing PN at commercially attractive levels. Moreover, the knowledge about the functions of target proteins of PLP is very important to understand how the B6 vitamer affects cellular metabolism upon overproduction.

## PLP-Dependent Proteins and Enzymes Involved in Vitamin B6 Metabolism in *Bacillus subtilis*

To identify the *B. subtilis* proteins and enzymes that require PLP for activity and are involved in vitamin B6 metabolism, we compared the Enzyme Commission (E.C.) numbers of the proteins from the *B. subtilis* 168 laboratory strain found in the *SubtiWiki* database (http://subtiwiki.uni-goettingen.de/v3/) (Zhu and Stülke, 2017) with the E.C. numbers that are deposited in the B6 database (Table 1) (http://bioinformatics.unipr.it/cgi-bin/bioinformatics/B6db/home.pl) (Percudani and Peracchi, 2009). We also describe proteins from the *SubtiWiki* database that are specific for *B. subtilis* and are therefore not present in the B6 database. Publications describing proteins involved in vitamin B6 metabolism in *B. subtilis* were also added to the Table. A recent mass spectrometry approach in combination with modified pyridoxal analogs identified proteins in the Gram-positive pathogen *Staphylococcus aureus* that probably depend on the B6 vitamer PLP (Hoegl et al., 2018). The study confirmed the binding of PLP to proteins of known and unknown function and identified 4 additional PLP-binding proteins (HemH, HemQ, YtoP, and YwlG) (see below). In total we ended up with 65 PLP-dependent proteins in *B. subtilis*, of which 61 proteins are *bona fide* PLP-dependent proteins. The PLP-dependency of four proteins remains to be experimentally validated. Table 1 also contains the PDB identifiers of structures that are available in the PDB database for the *B. subtilis* proteins. In case the structural information was not available, we have added the PDB identifiers from PLP-dependent homologs showing more than 27% overall sequence identity. We have also included information about the physiological functions of the proteins and their paralogs, the transcription factors that are involved in synthesis of the proteins and information about the sequence similarities with other proteins from the UniProt database (https://www.uniprot.org). The list of proteins involved in vitamin B6 metabolism in *B. subtilis* will certainly be extended in the future because PLP-dependent enzymes are ubiquitous and evolutionary diverse, making their classification based on sequence homology difficult.

**Table 1:** PLP-dependent proteins in *B. subtilis*.

**Protein**	**BSU no**.	**Essential^a^**	**E.C. no**.	**PDB no.^b^**	**Function**	**Pathway**	**Regulation**	**Paralogs/Protein familiy^c^**	**References**
**METABOLISM**									
**Amino acid biosynthesis**									
ArgD	BSU11220	No	2.6.1.11	2EH6 (*Aquifex aeolicus*, 45%)	Acetylornithine aminotransferase	Arginine biosynthesis	SigA, AhrC (–), CodY (–), YlxR (–)	PLP AAT class III family AAT, ArgD subfamily	Czaplewski et al., 1992; Brinsmade et al., 2014; Ogura and Kanesaki, 2018
AspB	BSU22370	No	2.6.1.1	1J32 (*Phosmidium lapideum*, 45%)	Aspartate aminotransferase	Aspartate biosynthesis	Unknown	AlaT (41%), PatA (41%)/PLP AAT class I family	Zhao et al., 2018
CysK	BSU00730	No	2.5.1.47	1Y7L (*Haemophilus influenzae*, 39%)	Cysteine synthase, control of CymR activity	Cysteine biosynthesis	SigA, SigM, Spx (+)	YtkP (59%), MccA (45%)/cysteine synthase, cystathione β-synthase family	Tanous et al., 2008
GlyA	BSU36900	No	2.1.2.1	2VI8 (*Geobacillus stearothermophilus*, 80%)	Serine hydroxymethyl- transferase	Glycine biosynthesis	SigA, T-Box, PurR (–)	SHMT family	Gutiérrez-Preciado et al., 2009
HisC	BSU22620	No	2.6.1.9	3FFH (*Listeria innocua*, 52%)	Histidinol-phosphate aminotransferase	Aromatic amino acids Biosynthesis	MtrB (–)	PLP AAT class II family	Nester and Montoya, 1976; Babitzke et al., 1992
IlvA	BSU21770	No	4.3.1.19	1TDJ (*E. coli*, 39%)	Threonine dehydratase	Branched-chain amino acid biosynthesis	CodY (–)	Ser/Thr dehydratase familiy	Molle et al., 2003a; Rosenberg et al., 2016
LysA	BSU23380	No	4.1.1.20	1HKW (*Mycobacterium tuberculosis*, 42%)	Diaminopimelate decarboxylase	Lysine biosynthesis	SigG, SpoVT (+)	Orn/Lys/Arg decarboxylase class II family	Kalcheva et al., 1997; Steil et al., 2005
MccA	BSU27260	No	–	4QL4 (*Bacillus anthracis*, 63%)	*O*-Acetylserine-thiol-lyase	Methionine/cysteine conversion	SigA, Spx (+), CymR (–)	CysK (45%), YtkP (42%)/cysteine synthase, cystathione β-synthase family	Nakano et al., 2003; Choi et al., 2006; Even et al., 2006
MccB	BSU27250	No	4.4.1.1	4L0O (*Helicobacter pylori*, 61%)	Cystathionine lyase/ homocysteine ⋎-lyase	Methionine/ cysteine conversion	SigA, Spx (+), CymR (–)	MetC (52%), MetI (48%)/Trans-sulfuration enzyme family	Nakano et al., 2003; Choi et al., 2006; Even et al., 2006
MetC	BSU11880	No	4.4.1.8	4L0O (*H. pylori*, 51%)	Cystathionine β-lyase	Methionine biosynthesis	SigA, S-box	MccB (52%), MetI (43%)/trans-sulfuration enzyme family	Grundy and Henkin, 1998; Auger et al., 2002; Tomsic et al., 2008
MetI	BSU11870	No	–	4L0O (*H. pylori*, 48%)	O-Succinyl-homoserine lyase	Methionine biosynthesis	SigA, S-box	MccB (48%), MetC (43%)/trans-sulfuration enzyme family	Grundy and Henkin, 1998; Auger et al., 2002; Tomsic et al., 2008
MtnE	BSU13580	No	–	2O1B (*Staphylococcus aureus*, 42%)	Glutamine transaminase	Methionine salvage	SigA, unknown	BacF (50%)/ PLP AAT class I family	Sekowska and Danchin, 2002; Berger et al., 2003
PatB	BSU31440	No	4.4.1.8	3T32 (*B. anthracis*, 46%)	Cystathionine β-lyase	Methionine biosynthesis	Unknown	PLP AAT class II family	Auger et al., 2005
SerC	BSU10020	No	2.6.1.52	1W23 (*Bacillus alcalophilus*, 59%)	3-Phosphoserine aminotransferase	Serine biosynthesis	Unknown	PLP AAT class V family	Sakai et al., 2002a
ThrC	BSU32250	No	4.2.3.1	1UIN (*Thermus thermophilus*, 51%)	Threonine synthase	Threonine biosynthesis	CodY (–), TnrA (–), ThrR (–)	Thr synthase family	Nicolas et al., 2012; Kriel et al., 2014; Mirouze et al., 2015; Rosenberg et al., 2016
TrpB	BSU22640	No	4.2.1.20	4NEG (*B. anthracis*, 60%)	Tryptophan synthase β-subunit	Tryptophan biosynthesis	MtrB (–)	TrpB family	Shimotsu et al., 1986; Babitzke et al., 1992
YbgE	BSU02390	No	2.6.1.42	3HT5 (*M. tuberculosis*, 48%)	Branched-chain amino acid aminotransferase	Branched-chain amino acid biosynthesis	CodY (–)	YwaA (60%)/PLP AAT class IV family	Molle et al., 2003a; Belitsky and Sonenshein, 2008, 2011
YwaA	BSU38550	No	2.6.1.42	3HT5 (*M. tuberculosis*, 42%)	Branched-chain amino acid aminotransferase	Branched-chain amino acid biosynthesis	CodY (–)	YbgE (60%)/PLP AAT class IV family	Kriel et al., 2014
**Amino acid catabolism**									
GabT	BSU03900	No	2.6.1.19	1SF2 (*E. coli*, 45%)	4-Aminobutyrate aminotransferase	4-Aminobutyrate utilization	SigA, GabR (+)	YhxA (%)/PLP AAT class III family	Belitsky and Sonenshein, 2002
GcvPA	BSU24560	No	1.4.4.2	1WYT (*Thermus thermophilus*, 56%)	Glycine decarboxylase subunit 1	Glycine utilization	Gly-box	GcvP family, N-terminal subuni family	Mandal et al., 2004
GcvPB	BSU24550	No	1.4.4.2	1WYT (*Thermus thermophilus*, 56%)	Glycine decarboxylase subunit 1	Glycine utilization	Gly-box	GcvP family, C-terminal subuni family	Mandal et al., 2004
Kbl	BSU17000	No	2.3.1.29	1FC4 (*E. coli*, 40%)	2-Amino-3-ketobutyrate CoA ligase	Threonine utilization	Unknown	BioF (45%)/ PLP AAT class II family	Nicolas et al., 2012
RocD	BSU40340	No	2.6.1.13	3RUY (*B. anthracis*, 76%)	Ornithine transaminase	Arginine, ornithine and citrulline utilization	SigL, Spo0A (–), CodY (–), AhrC (+), RocR (+)	PLP AAT class III family, OAT subfamily	Gardan et al., 1995; Molle et al., 2003a,b
**Antibacterial compounds**									
BacF	BSU37690	No	2.6.1.1	201B (*S. aureus*, 40%)	Aminotransferase	Bacilysin biosynthesis	AbrB (–), CodY (–), ScoC (–)	MtnE (50%)/ PLP AAT class I family	Inaoka et al., 2003, 2009; Karatas et al., 2003; Köroglu et al., 2011
NtdA	BSU10550	No	–	4K2I	3-Oxo-glucose-6-phosphate:glutamate aminotransferase	Kanosamine biosynthesis	NtdR (+)	DegT/DnrJ/EryC1 family	Inaoka et al., 2004; Inaoka and Ochi, 2011
**Iron metabolism**									
SufS	BSU32690	Yes	2.8.1.7	5J8Q	Cysteine desulfurase	Iron-sulfur cluster formation	SigA	PLP AAT class V family, Csd subfamily	Albrecht et al., 2010; Nicolas et al., 2012; Black and Dos Santos, 2015
**Carbon metabolism**									
GlgP	BSU30940	No	2.4.1.1	1PYG (*Oryctolagus cuniculus*, 45%)	Glycogen phosphorylase	Glycogen biosynthesis	SigE	Glycogen phosphorylase family	Kiel et al., 1994
**Nucleotide utilization**									
PucG	BSU32520	No	–	3ISL	*S*-Ureidoglycine-glyoxylate aminotransferase	Purine utilization	SigA, PucR (+)	PLP AAT class V family	Schultz et al., 2001; Beier et al., 2002; Ramazzina et al., 2010
**COFACTORS**									
**Biotin**									
BioA	BSU30230	No	2.6.1.62	3DRD	Lysine-8-amino-7-oxononanoate aminotransferase	Biotin biosynthesis	BirA (–)	YhxA (33%)/ PLP AAT class III family	Bower et al., 1996; Perkins et al., 1996
BioF	BSU30220	No	2.3.1.47	3A2B (*Sphingobacterium multivorum*, 37%)	8-Amino-7-oxononanoate synthase	Biotin biosynthesis	BirA (–)	Kbl (45%)/ PLP AAT class II family	Bower et al., 1996; Perkins et al., 1996
**Folate**									
PabC	BSU00760	No	4.1.3.38	4WHX (*Burkholderia pseudomallus*, 27%)	Aminodeoxy-chorismate lyase	Biosynthesis of folate	SigA, MtrB (–)	PLP AAT class IV family	de Saizieu et al., 1997
**Heme**									
GsaB	BSU08710	No	5.4.3.8	3BS8 (*B. subtilis*, 48%)	Glutamate-1-semialdehyde aminotransferase	Heme biosynthesis	Unknown	HemL (48%)/ PLP AAT class III family, HemL subfamily	Ge et al., 2010; Witzky et al., 2018
HemH^d^	BSU10130	No	4.99.1.1	2HK6	Coproporphyrin ferrochelatase	Heme biosynthesis	Unknown	Ferrochelatase family	Dailey et al., 2017
HemL	BSU28120	No	5.4.3.8	3BS8	Glutamate-1-semialdehyde aminotransferase	Heme biosynthesis	SigA, PerR (–)	GsaB (48%)/ PLP AAT class III family, HemL subfamily	Hansson et al., 1991; Herbig and Helmann, 2001; Ge et al., 2010
HemQ^d^	BSU37670	No	1.11.1-	5T2K (*Geobacillus stearothermophilus*, 67%)	Coproheme decarboxylase	Heme biosynthesis		UPF0447 family	Dailey et al., 2017
**NAD**									
NifS	BSU27880	No	2.8.1.7	4R5F (*Archaeoglobus fulgidus*, 34%)	Cysteine desulfurase	NAD biosynthesis	SigA, NadR (–)	YrvO (35%), NifZ (32%)/ PLP AAT class V family, NifS/IscS subfamily	Sun and Setlow, 1993; Rossolillo et al., 2005
**CELLULAR PROCESSES**									
**Cell wall metabolism**									
Alr	BSU04640	Yes	5.1.1.1	1L6G (*Geobacillus stearothermophilus*, 56%)	Alanine racemase	Peptidoglycan biosynthesis	Unknown	YncD (41%)/ Ala racemase family	Heaton et al., 1988; Nicolas et al., 2012
Dat	BSU09670	No	2.6.1.21	1G2W (*G. stearothermophilus*, 43%)	D-Alanine aminotransferase	Peptidoglycan biosynthesis	AbrB (–), CodY(–), ScoC (–)	PLP AAT class IV family	Nicolas et al., 2012
PatA	BSU14000	Yes	2.6.1.-	1GDE (*Pyrococcus* sp., 42%)	*N*-Acetyl-L,L-diaminopimelate aminotransferase	Biosynthesis of lysine and peptidoglycan	Unknown	AlaT (41%), AspB (41%)/PLP AAT class I family	Nicolas et al., 2012; Rueff et al., 2014
**INFORMATION PROCESSING**									
**tRNA modification**									
NifZ	BSU29590	No	–	1EG5 (*Thermotoga maritima*, 35%)	Cysteine desulfurase	4-Thiouridine in tRNA biosynthesis	Unknown	YrvO (42%), NifS (32%)/PLP AAT class V family, NifS/IscS subfamily	Nicolas et al., 2012; Rajakovich et al., 2012
YrvO	BSU27510	Yes	2.8.1.7	1EG5 (*T. maritima*, 35%)	Cysteine desulfurase	tRNA modification	Unknown	NifZ (42%), NifS (35%)/PLP AAT class V family, NifS/IscS subfamily	Nicolas et al., 2012; Black and Dos Santos, 2015
**Regulation of gene expression**									
GabR	BSU03890	No	–	4MGR	Regulator of *gabTD* and *gabR* genes	⋎-Aminobutyrate utilization	SigA, GabR (–)	MocR/GabR family; PLP AAT class I family (C-terminal section)	Belitsky and Sonenshein, 2002; Bramucci et al., 2011
**LIFESTYLES**									
**Sporulation**									
SpsC	BSU37890	No	–	1MDX (*Salmonella typhimurium*, 44%)	Aminotransferase	Spore coat polysaccharide synthesis	SigE, SigG, GerE (+)	DegT/DnrJ/EryC1 family	Eichenberger et al., 2004; Arrieta-Ortiz et al., 2015
YncD	BSU17640	No	5.1.1.1	1L6G (*Geobacillus stearothermophilus*, 42%)	Alanine racemase	Spore protection	SigE	Alr (41%)/Ala racemase family	Pierce et al., 2008
**Biofilm formation**									
EpsN	BSU34230	No	–	1O61 (*Campylobacter jejuni*, 46%)	UDP-2,6-Dideoxy 2-acetamido 4-keto glucose aminotransferase	N,N'-Diacetyl- bacillosamine biosynthesis	SigA, RemA (+), AbrB (–), SinR (–), EAR riboswitch	DegT/DnrJ/EryC1 family	Kearns et al., 2005; Irnov and Winkler, 2010; Marvasi et al., 2010; Chumsakul et al., 2011; Winkelman et al., 2013
SpeA	BSU14630	No	4.1.1.19	2X3L (*S. aureus*, 28%)	Arginine decarboxylase	Spermidine, polyamine biosynthesis	Unknown	YaaO (34%)/Orn/Lys/Arg decarboxylase class I family	Sekowska et al., 1998; Burrell et al., 2010; Nicolas et al., 2012
**POORLY CHARACTERIZED PROTEINS**									
**Regulation of gene expression**									
YcxD	BSU03560	No	–	4MGR (GabR, 27%)	Unknown	Unknown	Unknown	MocR/GabR family; PLP AAT class I family (C-terminal section)	Bramucci et al., 2011
YdeF	BSU05180	No	–	4MGR (GabR, 23%)	Unknown	Unknown	Unknown	MocR/GabR family; PLP AAT class I family (C-terminal section)	Bramucci et al., 2011
YdeL	BSU05240	No	–	4MGR (GabR, 23%)	Unknown	Unknown	Unknown	MocR/GabR family; PLP AAT class I family (C-terminal section)	Bramucci et al., 2011
YdfD	BSU05370	No	–	1WST (*Thermococcus profundus*, 30%)	Unknown	Unknown	Unknown	MocR/GabR family; PLP AAT class I family (C-terminal section)	Bramucci et al., 2011
YhdI	BSU09480	No	–	4MGR (GabR, 40%)	Unknown	Unknown	Unknown	MocR/GabR family; PLP AAT class I family (C-terminal section)	Bramucci et al., 2011
YisV	BSU10880	No	–	1WST (*Thermococcus profundus*, 29%)	Unknown	Unknown	Unknown	MocR/GabR family; PLP AAT class I family (C-terminal section)	Bramucci et al., 2011
**Putative enzymes**									
DsdA	BSU23770	No	4.3.1.18	3SS7 (*E. coli*, 58%)	D-Serine deaminase	Unknown	Unknown	Ser/Thr dehydratase familiy, DsdA subfamily	McFall, 1964; Nicolas et al., 2012; Urusova et al., 2012
KamA	BSU19690	No	5.4.3.2	2A5H (*Clostridium subterminale Sb4*, 60%)	Lysine 2,3-aminomutase	Unknown	SigE	Radical SAM superfamily, KamA family	Chen et al., 2000; Feucht et al., 2003
YaaO	BSU00270	No	4.1.1.19	2X3L (*S. aureus*, 36%)	Putative arginine decarboxylase	Control of Efp modification	SigK, SigW	SpeA (34%)/Orn/Lys/Arg decarboxylase class I family	Huang et al., 1999; Burrell et al., 2010; Nicolas et al., 2012; Witzky et al., 2018
YcbU	BSU02660	No	–	–	Putative cysteine desulfurase	Unknown	Unknown	PLP AAT class V family	Nicolas et al., 2012
YhdR	BSU09570	No	2.6.1.1	3ELE (*Eubacterium rectale*, 34%)	Putative aspartate aminotransferase	Unknown	Unknown	Unknown	Nicolas et al., 2012
YhxA	BSU09260	No	–	3N5M (*B. anthracis*, 64%)	Putative adenosylmethionine-8-amino-7-oxononanoate aminotransferase	Unknown	SigA	YodT (35%), BioA (33%), GabT (32%)/class III PLP AAT family	Holmberg et al., 1990; Richards et al., 2011
YlmE	BSU15380	No		1W8G (*E. coli*, 33%)	Unknown	PLP homeostasis, Ile and Val metabolism	Spo0A (–)	YggS/PROSC family	Molle et al., 2003b; Ito et al., 2013; Prunetti et al., 2016
YnbB	BSU17440	No	–	3JZL (*L. monocytogenes str. 4b f2365*, 67%)	Putative C-S lyase	Modification of Efp	Unknown	Unknown	Nicolas et al., 2012; Witzky et al., 2018
YodT	BSU19740	No	–	3I4J (*Deinococcus radiodurans*, 39%)	Putative adenosyl- methionine-8-amino-7-oxononanoate aminotransferase	Unknown	SigE	YhxA (35%), RocD (31%), ArgD (31%)/class III PLP AAT family	Feucht et al., 2003
YtkP	BSU29970	No	2.5.1.47	2EGU (*Geobacillus kaustophilus*, 57%)	Putative cysteine synthase	Unknown	Unknown	CysK (58%), MccA (42%)/cysteine synthase, cystathione β-synthase family	Nicolas et al., 2012
YtoP^d^	BSU29860	No	–	3KL9 (*Streptococcus pneumoniae*, 44% identity)	Putative glutamyl aminopeptidase	Unknown	Unknown	YsdC (48%)/ peptidase M42 family	Kim D. et al., 2010
YwlG^d^	BSU36910	No	–	1V8D (*T. thermophilus*, 48%)	Unknown	Affects Efp modification level	TnrA (–)	Unknown	Mirouze et al., 2015; Witzky et al., 2018
AlaT	BSU31400	No	–	1DJU (*Pyrococcus horikoshii*, 47%)	Methionine aminotransferase	Unknown	Unknown	AspB (41%), PatA (41%), class I PLP AAT family	Matsui et al., 2000; Nicolas et al., 2012

a *The proteins are essential for growth of B. subtilis in LB medium supplemented with glucose (Reuss et al., 2016)*.

b *The overall amino acid sequence identity to other proteins from B. subtilis is shown in brackets*.

c *The protein family according to UniProt (www.uniprot.org)*.

d *A large-scale mass spectrometry-based screen revealed that the proteins probably bind PLP in S. aureus (Hoegl et al., 2018). It has to be experimentally validated that HemH, HemQ, YtoP and YwlG are functional PLP-dependent proteins*.

## Functional Assignment of Known PLP-Dependent Proteins in *B. subtilis*

**Figure 2:**
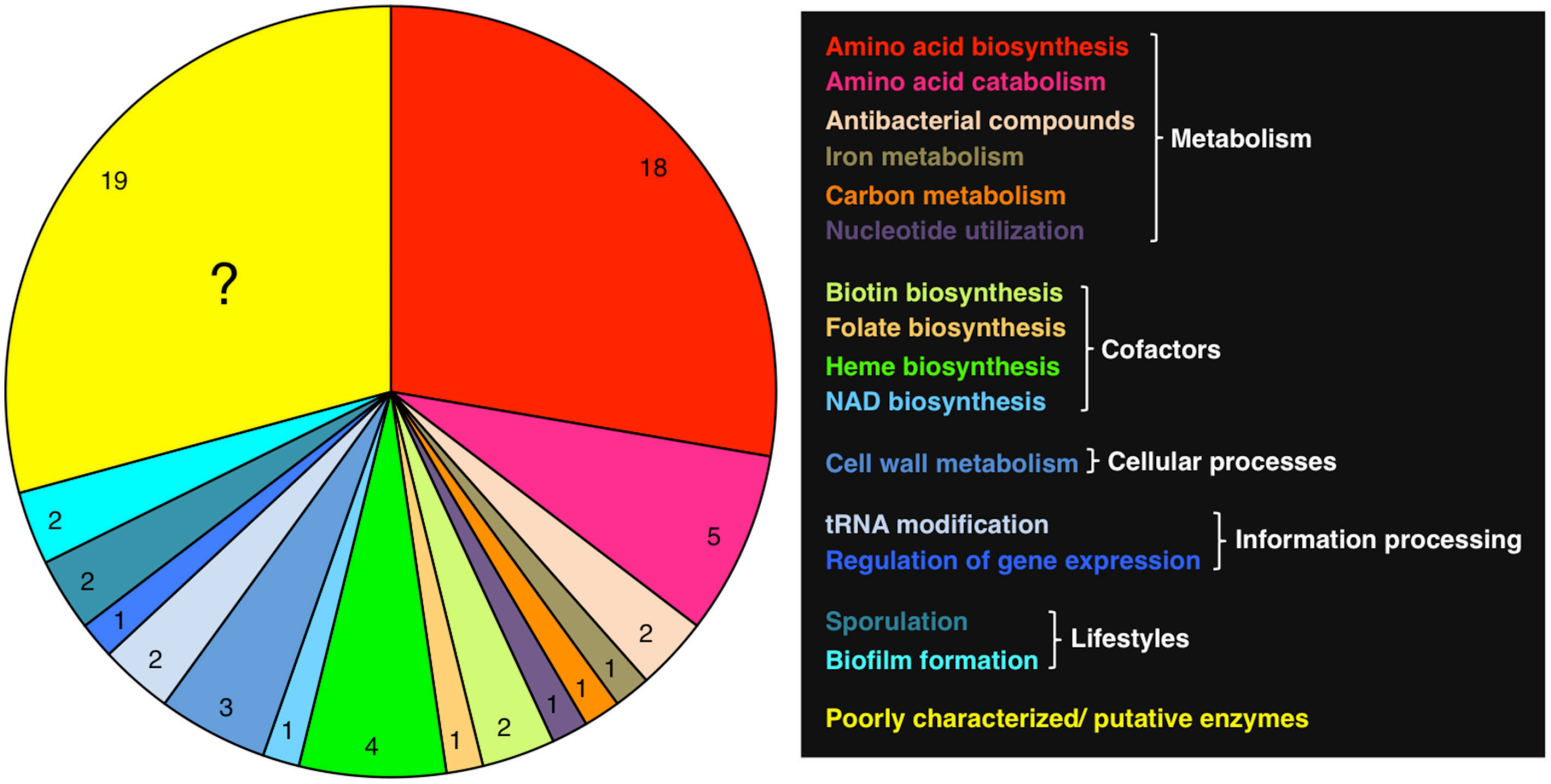
Functional distribution of PLP-dependent proteins in *B. subtilis*. See Table 1 for more information.

Most of the proteins that require PLP in *B. subtilis* are metabolic enzymes, of which the majority is involved in anabolism and catabolism of proteinogenic and non-proteinogenic amino acids (Table 1; Figure 2). The enzymes can be assigned to known protein families of PLP-dependent enzymes and for most of them it has been shown that they are indeed active in amino acid metabolism (Mehta et al., 1993; Mehta and Christen, 2000) (Table 1). *B. subtilis* also possesses a PLP-dependent 2-amino-3-ketobutyrate CoA ligase (Kbl), which could be involved in threonine utilization together with the L-threonine dehydrogenase Tdh (Schmidt et al., 2001; Reitzer, 2005). Both enzymes are encoded in the bicistronic *tdh-kbl* operon (Nicolas et al., 2012). The regulation of the *tdh* and *kbl* genes and the catalytic activities of the Tdh and Kbl enzymes remain to be studied. Two PLP-dependent enzymes BacF and NtdA are involved in the synthesis of bacilysin and kanosamine in *B. subtilis*. Bacilysin is a non-ribosomally synthesized peptide that is active against various bacteria and some fungi (Inaoka et al., 2003, 2009; Karatas et al., 2003; Köroglu et al., 2011). Kanosamine is an antibiotic, which is produced by *Bacillus* and *Streptomyces* species and inhibits cell wall synthesis in microorganisms (Dolak et al., 1980; Milner et al., 1996; Inaoka et al., 2004; Inaoka and Ochi, 2011; Vetter et al., 2013). The PLP-dependent enzymes SufS, GlgP, and PucG are involved in iron-sulfur cluster formation, glycogen biosynthesis, and purine utilization, respectively (Table 1). Homologs of SufS and GlgP are also present in *E. coli* (48 and 44% overall sequence identity, respectively). However, in *B. subtilis* the glycogen phosphorylase seems to be involved in a sporulation-specific process because the *glgP* gene is expressed early during sporulation in the mother cell (Kiel et al., 1994). Glycogen biosynthesis exclusively occurs in the presence of carbon sources allowing efficient sporulation (Kiel et al., 1994). Eight PLP-dependent enzymes are involved in the biosynthesis of the co-factors biotin, folate, heme and NAD (Table 1). While the biochemical and structural characterization of BioA, BioF, PabC, GsaB, HemL, and NifS revealed that the proteins require PLP for enzyme activity, it has to be investigated whether HemH and HemQ are *bona fide* PLP-dependent proteins. HemH and HemQ were recently identified in a mass spectrometry approach in the Gram-positive pathogen *Staphylococcus aureus* (Hoegl et al., 2018). *B. subtilis* possesses in total five PLP-dependent enzymes (Alr, Dat, PatA, NifZ, and YrvO) that are involved in cell wall metabolism and in information processing (Table 1). The alanine racemase Alr, the D-alanine aminotransferase Dat and the N-acetyl-L,L-diaminopimelate aminotransferase PatA generate precursors for the peptidoglycan of the cell wall. The tRNA-modifing enzymes NifZ and YrvO are both cysteine desulfurases that are active in biosynthesis of 4-thiouridine and 2-thiouridine, respectively, for the formation of modified tRNA molecules. YrvO transfers sulfur to the TrmU tRNA methyltransferase, which is essential for 2-thiouridine biosynthesis (Black and Dos Santos, 2015). Finally, four PLP-dependent enzymes play a role in sporulation and biofilm formation in *B. subtilis* (Table 1). While it has been shown that Sps proteins such as SpsC are required for spore germination (Cangiano et al., 2014), the role of the alanine racemase YncD in sporulation is currently unkown. The two biofilm-related enzymes, the arginine decarboxylases SpeA and the UDP-2,6-dideoxy-2-acetamido-4-keto-glucose aminotransferase EpsN are important for biosynthesis of polyamines such as spermidine and extracellular polysaccharides (Burrell et al., 2010; Marvasi et al., 2010). Indeed, *B. subtilis* strains lacking either SpeA or EpsN are defective in biofilm formation (Burrell et al., 2010; Pozsgai et al., 2012). SpeA also possesses a paralog (YaaO, 34% overall sequence identity), However, this proteins does not seem to be involved in biofilm formation (see below). To conclude, *B. subtilis* possesses several PLP-dependent enzymes that are involved in different cellular processes. Moreover, many PLP-dependent enzymes do have paralogs that have similar activities or fulfill specific functions in the cell, probably due to specialization during evolution (Table 1).

## PLP-Dependent Transcription Factors in *Bacillus subtilis*

*B. subtilis* possesses seven PLP-dependent DNA-binding transcription factors of which only one has been intensively characterized (see below; Table 1). The PLP-dependent transcription factors belong to the MocR-subfamily and contain a GntR-family DNA-binding domain at the N-terminus and an aminotransferase-like sensory domain at the C-terminus (Bramucci et al., 2011; Milano et al., 2015; Tramonti et al., 2015, 2017; Suvorova and Rodionov, 2016). The MocR-family-type PLP-dependent transcription factors that have been characterized are involved in controlling the expression of genes involved in PLP, γ-aminobutyrate, ecotoine, and taurine metabolism (Suvorova and Rodionov, 2016; Schulz et al., 2017; Tramonti et al., 2018). *B. subtilis* can utilize γ-aminobutyrate (GABA) as a source of nitrogen (Belitsky and Sonenshein, 2002). The catabolism of GABA requires the activities of the GABA aminotransferase GabT and the succinic semi-aldehyde dehydrogenase GabD that are encoded in the bicistronic *gabT-gabD* operon. The MocR-family-type regulator GabR activates the transcription of the *gabT* and *gabD* genes in a PLP- and GABA-dependent manner (Belitsky and Sonenshein, 2002; Belitsky, 2004a). The protein also binds to the *gabR* promoter and represses *gabR* transcription in the absence of GABA (Belitsky and Sonenshein, 2002; Edayathumangalam et al., 2013). Structual analysis of GabR revealed that the aminotransferase-like activity at the C-terminus of the protein is not essential for its function as a transcription regulator (Edayathumangalam et al., 2013). It has also been shown that the formation of an external aldimine between GABA and PLP causes a conformational transition from the open form to a closed form in the aminotransferase domain of GabR, triggering transcription activation of the *gabT-gabD* operon (Okuda et al., 2015a; Milano et al., 2017; Park et al., 2017; Wu et al., 2017). Moreover, the dimerization of the C-terminal domain of GabR enables the N-terminal domain to bind to DNA and the N-terminal domain controls the binding specificity of the effector domain (Okuda et al., 2015b). The analysis of the interaction between GabR and DNA revealed that two cognate binding sites and the bendability of the interjacent DNA sequence are important for transcription factor binding (Al-Zyoud et al., 2016; Amidani et al., 2017). To conclude, the biochemical and structural characterization of the *B. subtilis* GabR transcriptional regulator has uncovered mechanistic insights into the MocR-family-type PLP-dependent transcription factors and provides the basis for the characterization of the remaining six putative PLP-dependent DNA-binding transcription factors YcxD, YdeF, YdeL, YdfD, YhdI, and YisV from *B. subtilis*. Their characterization will presumably uncover novel metabolic pathways and transport systems (Suvorova and Rodionov, 2016).

## Poorly Characterized PLP-Dependent Enzymes

In addition to the 6 transcription factors whose DNA-binding activities depend on PLP and additional unknown effectors (see above), *B. subtilis* possesses several poorly characterized PLP-dependent enzymes (Table 1; Figure 2). The DsdA protein from *B. subtilis* shows about 58% overalls sequence identity with the *E. coli* D-serine deaminase (D-serine ammonia lyase) DsdA, which catalyzes the deamination of D-serine to form pyruvate and ammonia (Gale and Stephenson, 1938; McFall, 1964). The PLP-dependency and the structure of the enzyme from *E. coli* have been determined (Schnackerz et al., 1999; Urusova et al., 2012). Phylogenetic analyses suggest that the *E. coli* and *B. subtilis* D-serine deaminases and threonine synthases with similarities in the catalytic mechanisms may have evolved from a common ancestor (Parsot, 1986). The primary function of DsdA seems to be the detoxification of D-serine, which inhibits bacterial growth because it is a competitive antagonist of β-alanine in the pantothenate (vitamin B5) biosynthetic pathway, generating the precursor for coenzyme A biosynthesis (Cosloy and McFall, 1973). Previously, it has also been shown that *E. coli* mutants constitutively expressing DsdA are able to use D-serine as the sole source of carbon and nitrogen (Bloom and McFall, 1975). In *B. subtlilis* the *dsdA* gene is located in the *yqjP*-*yqjQ*-*dsdA*-*coaA*-*yqjT* operon, containing three genes of unknown function as well as the *dsdA* and *coaA* genes, of which the latter encodes the major pantothenate kinase CoaA (Ogata et al., 2014). The genetic context of the *dsdA* gene strongly suggests that the D-serine deaminase may be involved in the detoxification of D-serine that probably also interferes with pantothenate synthesis in *B. subtilis*. The presence of a D-serine deaminase may be explained by the fact that L-serine is more rapidly racemized than most other amino acids (Reitzer, 2005). It will be interesting to elucidate whether overproduction of DsdA allows *B. subtilis* to grow with D-serine as the sole source of carbon and nitrogen and whether DsdA is involved in the detoxification of D-serine.

The *B. subtilis* KamA enzyme shows about 60% overalls sequence identity with the PLP-, S-adenosyl-L-methionine and [4Fe-4S]-dependent lysine-2,3-aminomutase from the obligate anaerobe bacterium *Clostridium subterminale* (Table 1) (Lepore et al., 2005). The lysine-2,3-aminomutase catalyzes the converstion of L-lysine to L-β-lysine, which is the first step in the anaerobic degradation of lysine in clostridia (Chirpich et al., 1970). *In vitro* characterization of KamA from *B. subtilis* revealed that enzyme also catalyzes the conversion of L-lysine to L-β-lysine under anaerobic conditions (Chen et al., 2000). The KamA enzyme is only produced during sporulation of *B. subtilis* (Feucht et al., 2003). Therefore, the enzyme does not seem to play a role during vegetative growth. The lysine-2,3-aminomutase EpmB from *E. coli*, which shows about 31% overalls sequence identity with KamA from *B. subtilis*, has low lysine-2,3-aminomutase activity, indicating that L-lysine does not seem to be the natural substrate (Chen et al., 2000; Yanagisawa et al., 2010). Recently, it has been shown that the *E. coli* lysine-2,3-aminomutase EpmB enhances the lysylation of the elongation factor EF-P by the aminoacyl-tRNA synthetase GenX (Yanagisawa et al., 2010). The lysylation of EF-P is a post-translational modification that is essential for cell survival (Yanagisawa et al., 2010; Park et al., 2012). However, the physiological function of the lysine-2,3-aminomutase KamA from *B. subtilis* remains to be determined.

The *B. subtilis* YaaO enzyme, which belongs to class I Orn/Lys/Arg decarboxylases, encodes a putative arginine decarboxylase (Table 1). Arginine decarboxylases are important for biosynthesis of polyamines such as spermidine, substances that are crucial for biofilm formation (Burrell et al., 2010). The *B. subtilis* arginine decarboxylase SpeA, which can be considered as a paralog of YaaO (34% overall sequence identity), is indeed essential for the synthesis of polyamines and thus biofilm formation (see above) (Burrell et al., 2010). However, no biofilm-related phenotype has been reported so far for a *B. subtilis* mutant lacking YaaO. Recently, it has been reported that YaaO and two other proteins of unknown function (YfkA and YwlG, see below) influence the level of the post-translational aminopentanol modification of the elongation factor EF-P (Witzky et al., 2018). However, the precise role of YaaO in the modification of EF-P in *B. subtilis* remains to be elucidated.

The uncharacterized PLP-dependent proteins YcbU, AlaT, YhxA, and its paralog YodT are probably PLP-dependent amino acid transferases (Table 1). YcbU might be a cysteine desulfurase that is involved in co-factor biosynthesis (Mihara and Esaki, 2002). However, it remains to be elucidated whether YcbU is functional in *B. subtilis* because a mutant lacking YcbU shows no obvious phenotype (Koo et al., 2017). AlaT is similar to PLP-dependent methionine amino acid transferases and the protein shares about 47% overall sequence identity with an amino acid transferase from *Pyrococcus horikoshii* that acts on aromatic amino acids (Matsui et al., 2000). However, not experimental evidence supporting the annotation of AlaT is available. Both, YhxA and YodT are annotated as putative adenosylmethionine-8-amino-7-oxononanoate aminotransferases, enzymes that were shown to be involved in biotin biosynthesis in bacteria (Izumi et al., 1975). YhxA shares about 35 and 33% sequence identity with YodT and BioA, respectively. The PLP-dependent lysine-8-amino-7-oxononanoate aminotransferase BioA is required for biotin biosynthesis in *B. subtilis* (Table 1). Therefore, it is tempting to speculate that YhxA and YodT are also involved in biotin metabolism in this organism. The expression of the *yhxA* and *yodT* genes depends on SigA and on the sporulation-specific sigma factor SigE, respectively. Therefore, these enzymes seem to be active in different cellular differentiation processes of *B. subtilis*.

The *B. subtilis* YlmE protein of unknown function shows about 33% overall sequence identity with the YggS protein from *E. coli*. Recently, it has been shown that YggS is a PLP-binding protein, which belongs to a highly conserved COG0325 protein family and exists in almost all kingdoms of life, including bacteria, fungi and animals (Ito et al., 2013). The high conservation of YggS indicates that the protein fulfills an important function in bacteria. Indeed, the lack of YggS in *E. coli* affects balance of PLP homeostasis, sensitivity toward the B6 vitamer PN and perturbation of biosynthesis of branched-chain amino acids (Prunetti et al., 2016). Similar phenotypes have been associated to a mutant strain of *Synechococcus elongatus* PCC 7942 lacking the *pipY* gene, which encodes a COG0325 homolog (Labella et al., 2017; Tremiño et al., 2017). It will be very interesting to elucidate the precise function of COG0325 homologs in controlling vitamin B6 homeostasis.

The proteins YhdR, YnbB, and YwlG cannot be assigned to a specific protein family (Table 1). YhdR shares 34% overall sequence identity with an amino acid transferase from *Eubacterium rectale* but its role in amino acid metabolism is unknown (Table 1). Interestingly, like YaaO, YnbB, and YwlG are involved in the post-translational aminopentanol modification of the elongation factor EF-P (Witzky et al., 2018). While YwlG influences the level of the post-translational modification, YnbB seems to be required for the modification. The precise function of the proteins in controlling the activity of the elongation factor EF-P in *B. subtilis* needs further investigation. Moreover, it remains to be experimentally determined whether the function of YwlG depends on PLP.

The YtkP protein is a putative cysteine synthase that shares sequence similarity with the bifunctional cysteine synthase CysK and the *O-*acetylserine-thiol lyase MccA (Table 1). Since a *cysK mccA* double mutant is auxotrophic for cysteine, YtkP does not seem to be involved in cysteine biosynthesis. Thus, the function of YtkP remains elusive. The YtoP protein has been annotated as a putative glutamyl aminopeptidase because it shares about 44% overall sequence identity with PepA, a protease from *Streptococcus pneumoniae* that has been structurally and biochemically analyzed (Kim D. et al., 2010). Interestingly, YsdC, the paralog of YtoP (44% overall sequence identity), is annotated as an endo-1,4-β-glucanase (Table 1). Therefore, it is tempting to speculate whether YtoP is indeed involved in protein turnover. The binding of PLP to YtoP has to be experimentally validated. To conclude, *B. subtilis* contains several poorly characterized PLP-dependent proteins, which need to be studied in the future.

## Conclusions and Future Perspectives

For a complete understanding of the vitamin B6 metabolism of *B. subtilis* it is crucial to identify and characterize all the proteins that require the essential co-factor to fulfill their function. However, even for well-studied model bacteria like *B. subtilis* the complete set of the enzymes involved in vitamin B6 metabolism and the PLP-dependent proteins remains to be identified. Several bioinformatics-driven approaches have been performed to identify and classify PLP-dependent enzymes (Percudani and Peracchi, 2003, 2009). Even though the PLP-dependent proteins often show low sequence similarities, using sensitive Hidden Markov Model-base sequence similarity searches PLP-dependent proteins can be identified (Yoon, 2009). However, in case the protein has a fold that is different from the known PLP-dependent fold it is difficult to identify novel PLP-dependent proteins by sequence comparison. Even though structural similarity searches allowed assigning the PLP-dependent proteins to five distinct fold types (Mehta et al., 1993; Mehta and Christen, 2000; Catazaro et al., 2014), other approaches have to be pursued to uncover the full repertoire of PLP-dependent enzymes in a given organism. Indeed, mass spectrometry and biochemical approaches have been performed to identify proteins that were modified by PLP (Simon and Allison, 2009; Whittaker et al., 2015; Wu et al., 2018). As described above, a recent mass spectrometry approach identified proteins in the Gram-positive pathogen *S. aureus* that might depend on the B6 vitamer PLP (Hoegl et al., 2018). It will be interesting to evaluate whether the same approach will lead to the identification of novel PLP-dependent proteins in *B. subtilis* and related bacteria. Moreover, the transport systems for the uptake and export of the B6 vitamers PN and PL have to be identified by *B. subtilis*. The phosphatase involved in the dephosphorylation of PNP is so far unknown (Commichau et al., 2014, 2015). Furthermore, the function of the conserved YlmE protein (YggS in *E. coli*) in vitamin B6 homeostasis has to be studied. Finally, it has to be elucidated how the PLP molecules are delivered to their target proteins.

## Speciality Section

PLP-Dependent Enzymes: Extraordinary Versatile Catalysts and Ideal Biotechnological Tools for the Production of Unnatural Amino Acids and Related Compounds, in Process and Industrial Biotechnology, a section of the journal Frontiers in Bioengineering and Biotechnology.

## Author Contributions

BR, JR, and FC performed the database search. FC coordinated the work and wrote the manuscript with input from all authors.

### Conflict of Interest Statement

The authors declare that the research was conducted in the absence of any commercial or financial relationships that could be construed as a potential conflict of interest.
